# Retraction: The Tumor-Suppressive MicroRNA-135b Targets c-Myc in Osteoscarcoma

**DOI:** 10.1371/journal.pone.0282982

**Published:** 2023-03-29

**Authors:** The PLOS ONE Editors

The *PLOS ONE* Editors retract this article [1] because it was identified as one of a series of submissions for which we have concerns about peer review, authorship, and similarities across articles. Image similarities were noted between Fig 3B and Fig 5C in [1], and between Fig 1C in [1] and Fig 1D in [2, 3]. These concerns call into question the validity and provenance of the reported results. We regret that the issues were not addressed prior to the article’s publication.

All authors either did not respond directly or could not be reached.
